# Electric‐Field Control of Terahertz Response via Spin‐Corner‐Layer Coupling in Altermagnetic Bilayers

**DOI:** 10.1002/advs.202523285

**Published:** 2026-01-25

**Authors:** Jianhua Wang, Yilin Han, Shifeng Qian, Zhenxiang Cheng, Wenhong Wang, Zhi‐Ming Yu, Xiaotian Wang

**Affiliations:** ^1^ School of Material Science and Engineering Tiangong University Tianjin China; ^2^ Institute for Superconducting and Electronic Materials Faculty of Engineering and Information Sciences University of Wollongong Wollongong Australia; ^3^ Key Lab of Advanced Optoelectronic Quantum Architecture and Measurement (MOE) Beijing Key Lab of Nanophotonics & Ultrafine Optoelectronic Systems, and School of Physics Beijing Institute of Technology Beijing China; ^4^ Anhui Province Key Laboratory for Control and Applications of Optoelectronic Information Materials Department of Physics Anhui Normal University Wuhu Anhui China

**Keywords:** altermagnets, quantum dots, spin‐corner‐layer coupling, terahertz spintronics

## Abstract

Electric field control of electron charge and spin degrees of freedom is fundamental to modern semiconductor and spintronic devices. Yet controlling electromagnetic waves with an electric field, particularly in the terahertz (THz) band, remains a challenge. Here, we propose a spin‐corner‐layer coupling (SCLC) mechanism in second‐order topological altermagnetic bilayers. By using an electric field to influence electrons between different layers, the SCLC mechanism enables simultaneous control over corner and spin degrees of freedom, thereby allowing electric‐field tuning of the absorption, emission intensity, and even polarization of THz waves. Taking bilayer ${\mathrm{NiZrI}}_{6}$ nanodisks as a prototype, we demonstrate that an ultralow electrostatic field can switch both the spin and the layer polarizations of corner states. This dual switching modulates transition dipole moments and oscillator strengths between different corner states, thereby enabling the manipulation of THz waves. This study establishes a mechanism for the electric‐field control of spin and THz waves through SCLC, yielding important implications for the advancement of THz spintronics.

## Graphical abstract

A spin‐corner‐layer coupling mechanism is proposed in 2D altermagnetic bilayers. Exemplified by NiZrI6, the spin, layer, and topological corner states are simultaneously controlled by an ultralow electric field, enabling active modulation of terahertz absorption, emission, and polarization. This provides an efficient platform for compact terahertz spintronic devices such as modulators and integrated sensors.

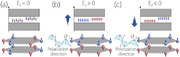

## 1 Introduction

Terahertz (THz) waves [1, 2, 3, 4, 5] have attracted significant attention due to their broad applications in high‐speed wireless communication [6], non‐intrusive standoff sensing [7, 8], and industrial quality control [9, 10]. Therefore, achieving effective control over THz waves holds great scientific significance and application value. Electric fields have long been established as the most direct and mature external tool in semiconductor electronics and spintronics, where they have been successfully used to manipulate charge and spin degrees of freedom (d.o.f.), forming the foundation of the entire information industry. Thus, a natural strategy is to channel electric‐field control of spin and related d.o.f. into controlling THz electromagnetic waves. Consistent with this strategy, the emerging field of THz spintronics [11, 12, 13, 14, 15, 16] exploits the electron's intrinsic spin to overcome the limitations of conventional electronics and optics and is now a rapidly expanding research frontier.

Recently, 2D second‐order topological insulators (SOTIs) [17, 18, 19, 20, 21, 22, 23, 24, 25, 26, 27, 28, 29] have attracted attention for their symmetry‐protected corner states, which localize at the intersection of two crystal edges. The nanodisks constructed from 2D SOTI act as quantum dots (QDs), possessing a new kind of d.o.f.—corner d.o.f. [30, 31, 32, 33]—which arises from the corner states and can be controlled by external fields [30, 34]. On the other hand, altermagnetic materials [35, 36, 37, 38, 39, 40, 41, 42, 43, 44, 45, 46, 47, 48, 49, 50, 51, 52, 53], distinct from conventional ferromagnets and antiferromagnets, have zero net magnetization but feature spin‐split electronic structures. Recent breakthroughs have further shown that electrical manipulation of spin d.o.f. in altermagnetic bilayers is feasible [54, 55, 56, 57], providing a platform for achieving electric‐field control of spin. Therefore, integrating the corner and layer d.o.f. of SOTI bilayer nanodisks with the intrinsic spin d.o.f. of altermagnets leads to altermagnetic bilayer SOTI nanodisks, a promising platform for electric‐field manipulation of spin and corners. Furthermore, corner‐state transitions may give rise to THz excitations with a pronounced polarization dependence, enabling efficient electric‐field control of the THz response.

Generally, low‐energy modes and elementary excitations in materials occur at different spatiotemporal scales and exist within the 0.1–10 THz range of the electromagnetic spectrum. Terahertz scanning near‐field optical microscopy (THz‐SNOM) [58, 59] overcomes the diffraction limit that constrains conventional far‐field THz spectroscopy, combining atomic force microscopy with pulsed THz sources. In recent years, experimental measurements have been performed on the THz response of various materials relative to substrates or other materials, such as soft materials [60, 61], the depth and effects of multilayer structures [62, 63], tissue characterization of cells [64], and near‐field plasmonic responses in topological insulators [65]. For 2D multilayer materials, Jing et al. [66] experimentally measured the low‐temperature nanoscale electromagnetic response of ${\mathrm{WTe}}_{2}$ with different layer numbers at THz frequencies, confirming a strong correlation between layer number and low‐temperature near‐field signal intensity. In the future, using THz‐SNOM, it is expected that the THz response in higher‐order topological materials under reversed external electric field control will be observed, further revealing its dependence on corner‐state transitions and THz excitations.

**FIGURE 1:**
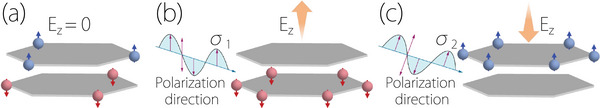
Schematic illustration of electric‐field control of terahertz response via SCLC in altermagnetic bilayer SOTI nanodisk. (a) In the absence of an out‐of‐plane electric field ($E_{z}$), the system exhibits intrinsic SCLC. (b,c) By reversing the direction of the $E_{z}$, the system exhibits dual switching of layer polarization ($P_{l}$) and spin polarization ($P_{s}$) at the corners, enabling electric‐field control of the absorption, emission intensity, and polarization of THz waves. $\sigma_{1}$ and $\sigma_{2}$ represent two different polarizations of THz waves.

In this work, we propose a new mechanism for controlling THz absorption and emission based on spin‐corner‐layer coupling (SCLC) in second‐order topological altermagnetic bilayers Figure 1. By using an electric field to influence electrons between different layers, this mechanism enables the simultaneous manipulation of both corner and spin d.o.f. Consequently, it allows for the control of the absorption, emission intensity, and even the polarization of THz waves. Through first‐principles calculations, we identify a bilayer ${\mathrm{NiZrI}}_{6}$ nanodisk—an intrinsic altermagnetic SOTI nanodisk—as a prototype platform exhibiting SCLC. The corners in ${\mathrm{NiZrI}}_{6}$ nanodisks, which exhibit both opposing spin polarization ($P_{s}$) and layer polarization ($P_{l}$), facilitate the interaction among corner, layer, and spin d.o.f. A small out‐of‐plane electric field triggers dual switching of spin and layer polarizations of the corners, thereby enabling electric‐field tuning of the THz responses. Our findings establish a viable route to electrically tunable THz spintronic platforms in 2D altermagnetic SOTI nanodisks.

## 2 Results and Discussion

### 2.1 SOTI Bilayer with Fractionally Quantized Corner Charge

**FIGURE 2:**
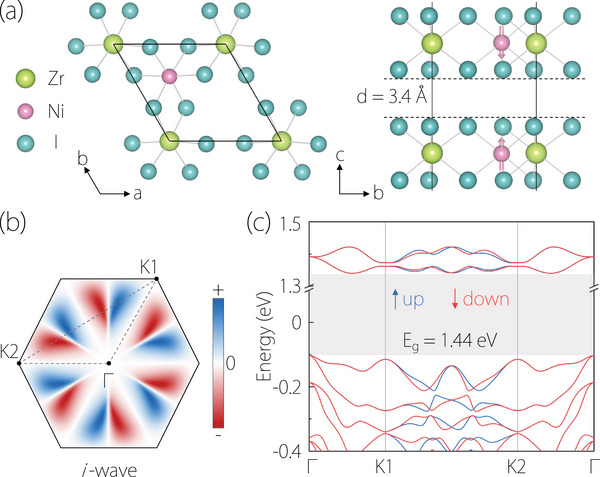
(a) Crystal structure of bilayer ${\mathrm{NiZrI}}_{6}$ with AA stacking, shown from different perspectives. (b) 2D surface Brillouin zone (BZ) and spin splitting of the valence band. (c) Spin‐polarized band structure of the altermagnetic bilayer ${\mathrm{NiZrI}}_{6}$ along the Γ‐K1‐K2‐Γ paths.

The bilayer ${\mathrm{NiZrI}}_{6}$, belongs to space group P312 (No. 149), is predicted to exhibit dynamic stability and possesses an interlayer antiferromagnetic structure (see Figure 2a) [54]. The optimized lattice constants are a = b = 7.19 Å, and the Wyckoff positions of Ni, Zr, and I atoms are 2h, 2g, and 6l, respectively. The spin‐polarized band structure for bilayer ${\mathrm{NiZrI}}_{6}$ without spin‐orbit coupling (SOC) is shown in Figure 2c, in which the spin splitting appears on the K1‐K2 path and a gap with the value of 1.44 eV occurs in both spin channels. Significantly, the in‐plane twofold rotation symmetry $C_{2∥}$ links two layers with opposing spins and is crucial for the emergence of i‐wave altermagnetism in the bilayer ${\mathrm{NiZrI}}_{6}$ (see Figure 2b). As shown in Figure S1 of the Supporting Information, the orbital‐resolved band structures of the 2D altermagnetic bilayer ${\mathrm{NiZrI}}_{6}$ are plotted within an energy window from –0.4 to 1.5 eV relative to the Fermi level. The two conduction bands immediately above the Fermi level are predominantly composed of Ni d‐orbitals, whereas the valence bands near the Fermi level are mainly derived from I p‐orbitals. In contrast, Zr atoms exhibit negligible orbital contributions across this energy range. The computational methods can be found in the Supporting Information.

Because the bilayer ${\mathrm{NiZrI}}_{6}$ perseveres threefold rotation‐symmetry $C_{3}$, and one can determine its topology by calculating the corner charge $Q_{\text{corner}\;}^{\left(3\right)}$ without considering the SOC, in terms of the rotation topological invariants, which shows as follow [67, 68, 69].

(1) $$Q_{1a}^{\left(3\right)}=\frac{\left|e\right|}{3}\left(n_{1a}^{\left(\mathrm{ion}\right)}-\nu -\left[K_{1}^{\left(3\right)}\right]-\left[K_{2}^{\left(3\right)}\right]-\left[K_{1}^{\prime (3)}\right]-\left[K_{2}^{\prime (3)}\right]\right),$$

where $n_{a}^{\left(\text{ion}\;\right)}$ is the ionic charges at Wyckoff position 1a, ν is the number of occupied bands. The summary of rotation topological invariants and corner charge $Q_{\text{corner}\;}^{\left(3\right)}$ are shown in Table 1. From it, one finds that the $Q_{\text{corner}\;}^{\left(3\right)}$ of a $C_{3}$‐preserved ${\mathrm{NiZrI}}_{6}$ nanodisk is $\frac{\left|e\right|}{3}$ for both spin‐up and spin‐down channels. The fractionally quantized corner charge reflects that the gaps in both spin channels are with nontrivial topology, and further, bilayer ${\mathrm{NiZrI}}_{6}$ is an i‐wave altermagnetic SOTI.

**TABLE 1:** The summary of rotation topological invariants and corner charge for altermagnetic bilayer ${\mathrm{NiZrI}}_{6}$ with and without SOC effects. Here, $\left[\Pi_{p}^{\left(3\right)}\right]=\#\Pi_{p}^{\left(3\right)}-\#\Gamma_{p}^{\left(3\right)}\;\left(p\;=\;1,\;2,\;3\right)$, which shows the difference between the number of states with $C_{3}$ eigenvalues at the Π and Γ points of the BZ.

	$n_{1a}^{\left(\mathrm{ion}\right)}$	ν	[$K_{1}^{\left(3\right)}$]	[ 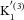 ]	[$K_{2}^{\left(3\right)}$]	[ 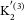 ]	$Q_{1a}^{\left(3\right)}$
Spin‐ up	12	64	1	1	−1	0	$\frac{\left|e\right|}{3}$
Spin‐down	12	64	1	1	−1	0	$\frac{\left|e\right|}{3}$
SOC	24	128	0	1	−1	−1	$\frac{2|e|}{3}$

### 2.2 Spin‐Layer‐Coupled and Direction‐Dependent Corners

**FIGURE 3:**
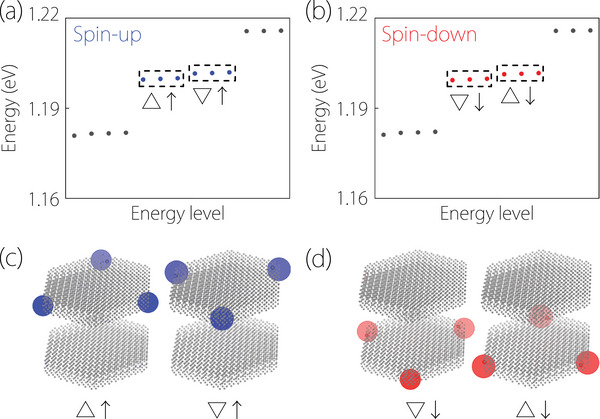
(a)‐(b) The energy spectrums for $C_{3}$‐preserved ${\mathrm{NiZrI}}_{6}$ nanodisk on a finite‐size hexagonal nanodisk, where four groups of threefold degenerate states (the blue and red dots) can be observed, calculated without SOC. (c)‐(d) The spatial distribution of the four groups of threefold degenerate states, termed as corners ▵↑, ▽↑, ▽↓, and ▵↓, respectively. The ▵ and ▽ show that the corners are direction‐dependent, and the ↑ and ↓ show that the corners have $P_{l}\left(P_{s}\right)>0$ and $P_{l}\left(P_{s}\right)<0$, respectively.

We come to study the corner states for the ${\mathrm{NiZrI}}_{6}$ nanodisk in both spin channels. We construct a Wannier tight‐binding model for a finite‐size hexagonal nanodisk based on $C_{3}$‐preserved bilayer ${\mathrm{NiZrI}}_{6}$. The energy spectrums of the hexagonal nanodisk calculated without SOC in both spin channels are shown in Figure 3. In Figure 3a, two groups of threefold degenerate states (blue dots) appear within the bulk and surface states (black dots). The spatial distributions for these two groups of states are plotted in Figure 3c. One can confirm that the threefold degenerate states are indeed corner states localized at three out of six corners of the finite‐size hexagonal nanodisk. Interestingly, these two groups of corners are direction‐dependent corners of the nanodisk, termed as corner ▵ and corner ▽, respectively.

Then, one can define the spin polarization and layer polarization of a corner state |ψ(k)⟩ as

(2) $$P_{s(l)}=\left\langle \psi \left(k\right)\right|{\hat{P}}_{s(l)}\left|\psi \left(k\right)\right\rangle ,$$

with ${\hat{P}}_{s}\equiv {\hat{s}}_{z}\left({\hat{P}}_{l}\equiv {\hat{r}}_{z}\right)$ denoting the z component of the spin (position) operator. $P_{l}>0\left(P_{s}>0\right)$ shows that |ψ(k)⟩ distributes more weight in the top layer (spin‐up), while $P_{l}<0\left(P_{s}<0\right)$ shows that |ψ(k)⟩ distributes more weight in the bottom layer (spin‐down). Hence, corner ▵ and corner ▽ in Figure 3c are completely distributed in spin‐up ($P_{s}>0$) and are situated in the top layer ($P_{l}>0$). They are therefore denoted as corner ▵↑ and corner ▽↑, respectively.

As shown in Figure 3b, the threefold degenerate states (red dots), i.e., the direction‐dependent corners ▽ and ▵, are also present within the bulk and surface states (black dots). The corner states in Figure 3b are totally distributed in spin‐down ($P_{s}<0$) and located in the bottom layer ($P_{l}<0$) (see Figure 3d), where they denote as corners ▽↓ and ▵↓. In ${\mathrm{NiZrI}}_{6}$ nanodisk, due to the altermagnetism, the direction‐dependent corners in two layers with opposite $P_{l}$ must come in pairs with opposite $P_{s}$, which is indicative of the nature of spin‐layer‐coupled corner states and results in the SCLC effect.

Note that the hexagonal nanodisk constructed from a ${\mathrm{NiZrI}}_{6}$ bilayer has a side length of 8a (a=0.72 nm), corresponding to an area of approximately $86.20\;{\mathrm{nm}}^{2}$. The SCLC effect remains robust against size reduction: when the side length is decreased from 8a to 4a (i.e., from $86.20\;{\mathrm{nm}}^{2}$ to $21.55\;{\mathrm{nm}}^{2}$), the spin‐layer‐coupled corner states still persist (see Figures S3 and S4 in the Supporting Information).

### 2.3 Electric‐Field‐Induced Corners With Dual‐Switchable $P_{l}$ and $P_{s}$

**FIGURE 4:**
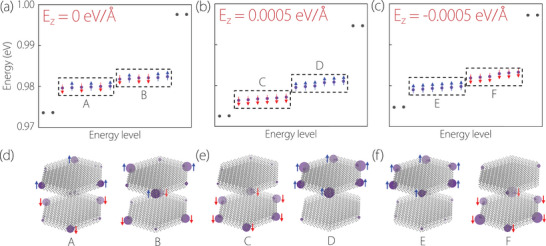
(a–c) The energy spectrums for $C_{3}$‐preserved ${\mathrm{NiZrI}}_{6}$ nanodisk with SOC for $E_{z}$ = 0, $E_{z}$ = 0.0005, and $E_{z}$ = ‐0.0005 eV/Å, respectively, in which six groups of sixfold degenerate states (A–F) can be observed. (d–f) The spatial distribution of the six groups of corner states, i.e., corners A‐F.

The inclusion of SOC results in spin‐split bands, such that states with the same momentum but opposite spins no longer remain degenerate in energy, i.e., E(k,↑)≠E(k,↓). Near the Fermi level in bilayer ${\mathrm{NiZrI}}_{6}$, the conduction bands are primarily formed by different d‐orbitals of the transition‐metal Ni atoms. More importantly, Ni is a heavy element, which enhances the SOC effect and shifts the conduction bands toward the Fermi level, reducing the band gap from 1.44 eV to 1.1 eV (Figure S5, Supporting Information)—a behavior similar to that observed in antiferromagnetic ${\mathrm{MnBi}}_{2}$ ${\mathrm{Te}}_{4}$ [70]. Notably, the SOTI nature and the spin‐layer‐coupled corners remain largely unchanged in ${\mathrm{NiZrI}}_{6}$ nanodisk when SOC is incorporated. As shown in Table 1, under SOC effect, the $Q_{\text{corner}\;}^{\left(3\right)}$ = $\frac{2|e|}{3}$ reflects the robustness of the SOTI nature. Figure 4a shows the energy spectrum ${\mathrm{NiZrI}}_{6}$ nanodisk with SOC and without electric fields. One finds that two groups of sixfold degenerate states (A and B states) appear, and their spatial distributions are exhibited in Figure 4d. Owing to the SOC, the ▵↑ and ▽↓ (▵↓ and ▽↑) corners with opposite $P_{l}$ $\left(P_{s}\right)$ coupled into corner A (B). By comparing with Figures 3c,d, and 4d, the spatial distribution of ▵↑ and ▽↓ (▵↓ and ▽↑) threefold degenerate states are almost the same with the spatial distribution of A (B) sixfold degenerate state, reflecting the robustness of the spin‐layer‐coupled corners in ${\mathrm{NiZrI}}_{6}$ nanodisk under SOC.

Corner A (B) can be roughly viewed as the merger of corner ▵↑ (▽↑) with $P_{l}$ $\left(P_{s}\right)$ >0 and corner ▽↓ (▵↓) with $P_{l}$ $\left(P_{s}\right)$ <0, as illustrated in Figure 4d. Interestingly, we can anticipate the realization of an electric‐field‐induced switchable sign of $P_{l}\left(P_{s}\right)$ for the corners as a result of the SCLC and direction‐dependent corners. Under a positive electric field, such as $E_{z}$ = 0.0005 eV/Å, the corner ▵↑ (▵↓) in corner A (B) changes to the corner ▵↓ (▵↑), as illustrated in Figure 4b,e. Conversely, the corner ▽↓ (▽↑) in corner A (B) has been inverted to the corner ▽↑ (▽↓) under a negative electric field, such as $E_{z}$ = ‐0.0005 eV/Å(see Figure 4c,f).

Hence, one can conclude that the signs of $P_{l}$ and $P_{s}$ for the direction‐dependent corners ▵ and ▽ can be dual‐switched by reversing the electric field. The reasons can be explained as follows: Spin‐layer locking enables the modulation of layers via electric fields, thereby controlling the spin orientation. The electric field breaks the $C_{2∥}$ symmetry: a positive (upward) electric field raises the energy of the corner states in the upper layer, and a negative (downward) electric field elevates the energy of the corner states in the lower layer. As a result, the spin for the corners experiences a corresponding reversal in alignment with the reversal of the layer for the corners.

### 2.4 Electric‐Field Tuning of the THz Response

The corner‐state energy gap of ${\mathrm{NiZrI}}_{6}$ nanodisk, a type of QD, is only a few meV, indicating that it can correspond to electromagnetic waves in the THz frequency range. Such behavior is unique to corner‐state QDs, whereas conventional nanometer‐sized QDs usually exhibit gaps much larger than the THz energy scale [71].

In spectroscopy, the THz responses can be quantitatively described by the absorbance. The absorbance $f_{12}$ of a transition from a state $|\psi_{1}\rangle$ to a state $|\psi_{2}\rangle$ is defined as

(3) $$f_{12}=\frac{2}{3}\frac{m_{e}}{\hbar^{2}}\left(E_{2}-E_{1}\right)\sum_{\alpha =x,y,z}{\left|\langle \psi_{1}\left|R_{\alpha }\right|\psi_{2}\rangle \right|}^{2},$$

where $m_{e}$ is the mass of an electron and ℏ is the reduced Planck constant. $E_{1}$ and $E_{2}$ are the energies of states $|\psi_{1}\rangle$ and $|\psi_{2}\rangle$, respectively. The $\langle \psi_{1}\left|R_{\alpha }\right|\psi_{2}\rangle$ is the transition dipole moment of two states, where $R_{\alpha }$ is the position operator of α direction. Here, we only consider the absorbance between the corner states since the transitions between other states are out of the range of THz frequency. To simulate the experimentally observed absorption spectrum, we apply broadening to the theoretically obtained transition data, resulting in absorbances manifested as a series of absorption peaks.

**FIGURE 5:**
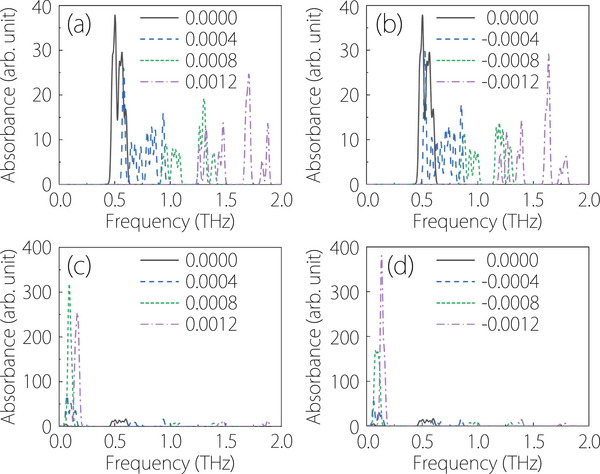
(a,b) Absorption spectrum when the highest occupied state is at the sixth corner state. (c,d) Absorption spectrum when the highest occupied state is at the third corner state. Black solid lines denote the case without an electric field, whereas colored dashed lines represent the cases with different values of positive (a, c) and negative (b, d) electric fields.

We first consider the scenario where electrons occupy half (one‐sixth) of the 12 available corner states. The absorption peak is observed near 0.5 THz, as shown in Figure 5a,b. In this configuration, both the occupied and unoccupied corner states are distributed across the upper and lower layers Figure 4d. This allows for intralayer transitions, resulting in a large oscillator strength and a prominent absorption peak. However, when an upward or downward electric field is applied, the occupied corner states become exclusively distributed in either the upper or lower layer, while the unoccupied states are confined to the opposite layer [Figure 4e,f, and Figure S6 (Supporting Information)]. This spatial separation suppresses intralayer transitions, leading to a significant reduction in oscillator strength and a substantial decrease in the absorption peak's intensity. Concurrently, the increasing energy gap between the occupied and unoccupied corner states causes a blueshift of the absorption peak. At an electric field strength of ± 0.0012 eV/Å, the peak shifts to nearly 2 THz, as depicted in Figure 5a,b. The studies [72, 73, 74, 75] consistently employed electric fields exceeding 0.1 V/Å (e.g., ∼0.10 V/Å in ${\mathrm{CrI}}_{3}$ [73], ∼0.11–0.185 V/Å in ${\mathrm{Na}}_{3}\mathrm{Bi}$ [74], and ∼0.142 V/Å in 1T'‐${\mathrm{MoS}}_{2}$ [75]). These fields are significantly stronger than those applied in our work.

Next, we examine the scenario where electrons occupy one‐third of the corner states. The absorption peak remains at 0.5 THz, as shown in Figure 5c,d. In contrast to the half‐filling case, the application of an electric field produces the opposite effect. The absorption peak intensity increases significantly, while its position redshifts to approximately 0.1 THz Figure 5c,d. This occurs because the occupied and unoccupied corner states remain distributed within the same layer [Figure 4e,f; Figure S6 (Supporting Information)]. For instance, with an upward electric field (positive value), the three occupied corner states are all in the lower layer, along with three of the unoccupied states. This co‐location enables intralayer transitions, leading to a notable enhancement of the absorption peak. Simultaneously, the reduced energy gap between the occupied and unoccupied states causes the observed redshift. Furthermore, with an upward electric field, the absorption peak is primarily contributed by spin‐down electrons, whereas a downward electric field leads to a contribution from spin‐up electrons (Figure S6 in the Supporting Information). Therefore, while the position and intensity of the absorption peak remain relatively unchanged when the electric field direction is reversed, the different spins contributing to the absorption result in a change in the direction of the transition dipole moment (Table S1 in the Supporting Information). This allows the material to exhibit a different absorption capability for different polarizations of THz electromagnetic waves when the electric field is switched.

## 3 Summary

In this work, we propose a new concept of coupling among corner, spin, and layer d.o.f., named SCLC. The SCLC originates from the spin‐layer‐coupled corner states, distinguished by corners exhibiting opposing $P_{l}$ and $P_{s}$. Based on a first‐principle calculation, we predict that the nanodisk constructed from altermagnetic bilayer ${\mathrm{NiZrI}}_{6}$ with AA stacking is the first platform with SCLC. Due to the SCLC in the ${\mathrm{NiZrI}}_{6}$ nanodisk, reversing the direction of the external electric field can dual‐switch the signs of $P_{l}$ and $P_{s}$ of the corners, thereby enabling effective control of THz response. The SCLC effect remains robust under size reduction, and the ${\mathrm{NiZrI}}_{6}$ nanodisk operates entirely at the nanometer scale, offering a compact and electrically driven platform for THz spintronics. This substantial contribution has the potential to significantly advance the field of 2D SOTIs, altermagents, corner‐state QDs, and multi‐d.o.f. couplings, as well as the development and application of nanoscale THz spintronic platforms.

## Conflicts of Interest

The authors declare no conflicts of interest.

## Supporting information




**Supporting File**: advs74046‐sup‐0001‐SuppMat.pdf.

## Data Availability

The data that support the findings of this study are available from the corresponding author upon reasonable request.

## References

[1] H. Hamster , A. Sullivan , S. Gordon , W. White , and R. W. Falcone , “Subpicosecond, Electromagnetic Pulses from Intense Laser‐Plasma Interaction,” Physical Review Letters 71 (1993): 2725. 10054760 10.1103/PhysRevLett.71.2725

[2] H. Hamster , A. Sullivan , S. Gordon , and R. W. Falcone , “Short‐Pulse Terahertz Radiation from High‐Intensity‐Laser‐Produced Plasmas,” Physical Review E 49 (1994): 671. 10.1103/physreve.49.671 9961261

[3] X. Xie , J. Dai , and X.‐C. Zhang , “Coherent Control of THz Wave Generation in Ambient Air,” Physical Review Letters 96 (2006): 075005. 16606102 10.1103/PhysRevLett.96.075005

[4] D. Zhang , Z. Lü , C. Meng , et al., “Synchronizing Terahertz Wave Generation with Attosecond Bursts,” Physical Review Letters 109 (2012): 243002. 23368313 10.1103/PhysRevLett.109.243002

[5] J. Shi , D. Yoo , F. Vidal‐Codina , et al., “A Room‐Temperature Polarization‐Sensitive CMOS Terahertz Camera Based on Quantum‐Dot‐Enhanced Terahertz‐to‐Visible Photon Upconversion,”Nature Nanotechnology 17 (2022): 1288. 10.1038/s41565-022-01243-9 36329270

[6] J. Ma , R. Shrestha , J. Adelberg , et al., “Security and Eavesdropping in Terahertz Wireless Links,” Nature 563 (2018): 89. 30323288 10.1038/s41586-018-0609-x

[7] S.‐H. Ding , Q. Li , R. Yao , and Q. Wang , “High‐Resolution Terahertz Reflective Imaging and Image Restoration,” Applied Optics 49 (2010): 6834. 21173813 10.1364/AO.49.006834

[8] J. Liu , J. Dai , S. L. Chin , and X.‐C. Zhang , “Broadband Terahertz Wave Remote Sensing Using Coherent Manipulation of Fluorescence from Asymmetrically Ionized Gases,” Nature Photonics 4 (2010): 627.

[9] F. Rutz , M. Koch , S. Khare , M. Moneke , H. Richter , and U. Ewert , “Terahertz Quality Control of Polymeric Products,” International Journal of Infrared and Millimeter Waves 27 (2006): 547.

[10] I. Duling and D. Zimdars , “Revealing Hidden Defects,” Nature Photonics 3 (2009): 630.

[11] T. Kampfrath , M. Battiato , P. Maldonado , et al., “Terahertz Spin Current Pulses Controlled by Magnetic Heterostructures,” Nature Nanotechnology 8 (2013): 256. 10.1038/nnano.2013.43 23542903

[12] T. Seifert , S. Jaiswal , U. Martens , et al., “Efficient Metallic Spintronic Emitters of Ultrabroadband Terahertz Radiation,”Nature Photonics 10 (2016): 483.

[13] J. Walowski and M. Münzenberg , “Perspective: Ultrafast Magnetism and THz Spintronics,” Journal of Applied Physics 120 (2016): 140901.

[14] Y. Wu , M. Elyasi , X. Qiu , et al., “High‐Performance THz Emitters Based on Ferromagnetic/Nonmagnetic Heterostructures,” Advanced Materials 29 (2017): 1603031. 10.1002/adma.201603031 27885714

[15] P. Agarwal , L. Huang , S. Ter Lim , and R. Singh , “Electric‐Field Control of Nonlinear THz Spintronic Emitters,” Nature Communications 13 (2022): 4072. 10.1038/s41467-022-31789-0 PMC9283400 35835753

[16] I. Ilyakov , A. Brataas , T. V. A. G. de Oliveira , et al., “Efficient Ultrafast Field‐Driven Spin Current Generation for Spintronic Terahertz Frequency Conversion,” Nature Communications 14 (2023): 7010. 10.1038/s41467-023-42845-8 PMC10622539 37919284

[17] F. Zhang , C. L. Kane , and E. J. Mele , “Topological Mirror Superconductivity,” Physical Review Letters 111 (2013): 056403. 23952424 10.1103/PhysRevLett.111.056403

[18] W. A. Benalcazar , B. A. Bernevig , and T. L. Hughes , “Quantized Electric Multipole Insulators,” Science 357 (2017): 61. 28684520 10.1126/science.aah6442

[19] J. Langbehn , Y. Peng , L. Trifunovic , F. von Oppen , and P. W. Brouwer , “Reflection‐Symmetric Second‐Order Topological Insulators and Superconductors,”Physical Review Letters 119 (2017): 246401. 29286744 10.1103/PhysRevLett.119.246401

[20] W. A. Benalcazar , B. A. Bernevig , and T. L. Hughes , “Electric Multipole Moments, Topological Multipole Moment Pumping, and Chiral Hinge States in Crystalline Insulators,” Physical Review B 96 (2017): 245115.

[21] M. Ezawa , “Higher‐Order Topological Insulators and Semimetals on the Breathing Kagome and Pyrochlore Lattices,” Physical Review Letters 120 (2018): 026801. 29376716 10.1103/PhysRevLett.120.026801

[22] C. W. Peterson , W. A. Benalcazar , T. L. Hughes , and G. Bahl , “A Quantized Microwave Quadrupole Insulator with Topologically Protected Corner States,” Nature 555 (2018): 346. 29542690 10.1038/nature25777

[23] X.‐L. Sheng , C. Chen , H. Liu , et al., “Two‐Dimensional Second‐Order Topological Insulator in Graphdiyne,” Physical Review Letters 123 (2019): 256402. 31922761 10.1103/PhysRevLett.123.256402

[24] M. J. Park , Y. Kim , G. Y. Cho , and S. Lee , “Higher‐Order Topological Insulator in Twisted Bilayer Graphene,” Physical Review Letters 123 (2019): 216803. 31809156 10.1103/PhysRevLett.123.216803

[25] R. Chen , C.‐Z. Chen , J.‐H. Gao , B. Zhou , and D.‐H. Xu , “Higher‐Order Topological Insulators in Quasicrystals,” Physical Review Letters 124 (2020): 036803. 32031860 10.1103/PhysRevLett.124.036803

[26] C. Chen , Z. Song , J.‐Z. Zhao , et al., “Universal Approach to Magnetic Second‐Order Topological Insulator,” Physical Review Letters 125 (2020): 056402. 32794859 10.1103/PhysRevLett.125.056402

[27] E. Lee , R. Kim , J. Ahn , and B.‐J. Yang , “Two‐Dimensional Higher‐Order Topology in Monolayer Graphdiyne,” npj Quantum Mater 5 (2020): 1.

[28] S. Qian , C.‐C. Liu , and Y. Yao , “Second‐Order Topological Insulator State in Hexagonal Lattices and Its Abundant Material Candidates,” Physical Review B 104 (2021): 245427.

[29] S. Qian , G.‐B. Liu , C.‐C. Liu , and Y. Yao , “C_n_‐Symmetric Higher‐Order Topological Crystalline Insulators in Atomically Thin Transition Metal Dichalcogenides,” Physical Review B 105 (2022): 045417.

[30] Y. Han , C. Cui , X.‐P. Li , et al., “Cornertronics in Two‐Dimensional Second‐Order Topological Insulators,” Physical Review Letters 133 (2024): 176602. 39530811 10.1103/PhysRevLett.133.176602

[31] J. Gong , Y. Wang , Y. Han , et al., “Hidden Real Topology and Unusual Magnetoelectric Responses in Two‐Dimensional Antiferromagnets,” Advanced Materials 36 (2024): 2402232. 10.1002/adma.202402232 38684179

[32] Y. Han , T. He , R.‐W. Zhang , Z. Li , Z.‐M. Yu , and Y. Yao , “Real Chern Insulator in Monolayer Decorated Transition Metal Nitrides,” Advanced Functional Materials n/a (2025): 2505282.

[33] J. Wang , X. Yang , Z. Yang , et al., “Pentagonal 2D Altermagnets: Material Screening and Altermagnetic Tunneling Junction Device Application,” Advanced Functional Materials n/a (2025): 2505145.

[34] R. Li , X. Zou , Y. Bai , et al., “Layer‐Coupled Corner States in Two‐Dimensional Topological Multiferroics,” Materials Horizons 11 (2024): 2242. 38421336 10.1039/d3mh01266b

[35] L. Šmejkal , J. Sinova , and T. Jungwirth , “Emerging Research Landscape of Altermagnetism,” Physical Review X 12 (2022): 040501.

[36] Z. Feng , X. Zhou , L. Šmejkal , et al., “An Anomalous Hall Effect in Altermagnetic Ruthenium Dioxide,” Nature Electronics 5 (2022): 735.

[37] J. Krempaský , L. Šmejkal , S. W. D'Souza , et al., “Altermagnetic Lifting of Kramers Spin Degeneracy,” Nature 626 (2024): 517. 38356066 10.1038/s41586-023-06907-7 PMC10866710

[38] L. Bai , W. Feng , S. Liu , L. Šmejkal , Y. Mokrousov , and Y. Yao , “Altermagnetism: Exploring New Frontiers in Magnetism and Spintronics,” Advanced Functional Materials 34 (2024): 2409327.

[39] S. Lee , S. Lee , S. Jung , et al., “Broken Kramers Degeneracy in Altermagnetic MnTe,” Physical Review Letters 132 (2024): 036702. 38307068 10.1103/PhysRevLett.132.036702

[40] O. Fedchenko , J. Minár , A. Akashdeep , et al., “Observation of Time‐Reversal Symmetry Breaking in the Band Structure of Altermagnetic RuO_2_ ,” Science Advances 10 (2024): eadj4883. 38295181 10.1126/sciadv.adj4883 PMC10830110

[41] Z. Guo , X. Wang , W. Wang , G. Zhang , X. Zhou , and Z. Cheng , “Spin‐Polarized Antiferromagnets for Spintronics,” Advanced Materials 37 (2025): 2505779. 40534322 10.1002/adma.202505779 PMC12422093

[42] S. Bhowal and N. A. Spaldin , “Ferroically Ordered Magnetic Octupoles in d‐Wave Altermagnets,” Physical Review X 14 (2024): 011019.

[43] X. Zhou , W. Feng , R.‐W. Zhang , et al., “Crystal Thermal Transport in Altermagnetic RuO_2_ ,” Physical Review Letters 132 (2024): 056701. 38364129 10.1103/PhysRevLett.132.056701

[44] Y. Liu , J. Yu , and C.‐C. Liu , “Twisted Magnetic Van der Waals Bilayers: An Ideal Platform for Altermagnetism,” Physical Review Letters 133 (2024): 206702. 39626711 10.1103/PhysRevLett.133.206702

[45] C. Song , H. Bai , Z. Zhou , et al., “Altermagnets as a New Class of Functional Materials,” Nature Reviews Materials 10 (2025): 473.

[46] Z. Zhou , X. Cheng , M. Hu , et al., “Manipulation of the Altermagnetic Order in CrSb via Crystal Symmetry,” Nature 638 (2025): 645. 39939768 10.1038/s41586-024-08436-3

[47] X. Duan , J. Zhang , Z. Zhu , et al., “Antiferroelectric Altermagnets: Antiferroelectricity Alters Magnets,” Physical Review Letters 134 (2025): 106801. 40153648 10.1103/PhysRevLett.134.106801

[48] M. Gu , Y. Liu , H. Zhu , et al., “Ferroelectric Switchable Altermagnetism,” Physical Review Letters 134 (2025): 106802. 40153660 10.1103/PhysRevLett.134.106802

[49] W. Sun , C. Yang , W. Wang , et al., “Proposing Altermagnetic‐Ferroelectric Type‐III Multiferroics with Robust Magnetoelectric Coupling,” Advanced Materials 37 (2025): 2502575. 40211656 10.1002/adma.202502575 PMC12232234

[50] Z. Li , Z. Zhang , Y. Chen , et al., “Fully Field‐Free Spin‐Orbit Torque Switching Induced by Spin Splitting Effect in Altermagnetic RuO_2_ ,” Advanced Materials 37 (2025): 2416712. 10.1002/adma.202416712 39967356

[51] H. Chen , Z.‐A. Wang , P. Qin , et al., “Spin‐Splitting Magnetoresistance in Altermagnetic RuO_2_ Thin Films,” Advanced Materials 37 (2025): 2507764. 10.1002/adma.202507764 40492877

[52] B. Jiang , M. Hu , J. Bai , et al., “A Metallic Room‐Temperature d‐Wave Altermagnet,”Nature Physics 21 (2025): 754.

[53] H. Zeng , W. Zhang , C. Qiu , D.‐Z. Ding , and J. Zhao , “Symmetry Breaking Induced Nonrelativistic Spin Splitting and Spontaneous Valley Polarization in Altermagnetic Ca(CoN)_2_ Bilayer,” Applied Physics Letters 126 (2025): 202405.

[54] B. Pan , P. Zhou , P. Lyu , H. Xiao , X. Yang , and L. Sun , “General Stacking Theory for Altermagnetism in Bilayer Systems,” Physical Review Letters 133 (2024): 166701. 39485963 10.1103/PhysRevLett.133.166701

[55] R.‐W. Zhang , C. Cui , R. Li , et al., “Predictable Gate‐Field Control of Spin in Altermagnets with Spin‐Layer Coupling,” Physical Review Letters 133 (2024): 056401. 39159119 10.1103/PhysRevLett.133.056401

[56] Y. Che , H. Lv , X. Wu , and J. Yang , “Bilayer Metal–Organic Framework Altermagnets with Electrically Tunable Spin‐Split Valleys,”Journal of the American Chemical Society 147 (2025): 14806. 40251739 10.1021/jacs.5c04106

[57] Y. Zhu , M. Gu , Y. Liu , et al., “Sliding Ferroelectric Control of Unconventional Magnetism in Stacked Bilayers,”Physical Review Letters 135 (2025): 056801. 40824793 10.1103/dmzg-ck2t

[58] H. T. Stinson , A. Sternbach , O. Najera , et al., “Imaging the Nanoscale Phase Separation in Vanadium Dioxide Thin Films at Terahertz Frequencies,” Nature Communications 9 (2018): 3604. 10.1038/s41467-018-05998-5 PMC6127259 30190517

[59] J. Zhang , X. Chen , S. Mills , et al., “Terahertz Nanoimaging of Graphene,” ACS Photonics 5 (2018): 2645.

[60] B. T. O'Callahan , K.‐D. Park , I. V. Novikova , et al., “In Liquid Infrared Scattering Scanning Near‐Field Optical Microscopy for Chemical and Biological Nanoimaging,” Nano Letters 20 (2020): 4497. 32356991 10.1021/acs.nanolett.0c01291

[61] X. Zhao , D. Li , Y.‐H. Lu , et al., “In Vitro Investigation of Protein Assembly by Combined Microscopy and Infrared Spectroscopy at the Nanometer Scale,” Proceedings of the National Academy of Sciences of the United States of America 119 (2022): e2200019119. 35914130 10.1073/pnas.2200019119 PMC9371722

[62] X. Guo , X. He , Z. Degnan , et al., “Terahertz Nanospectroscopy of Plasmon Polaritons for the Evaluation of Doping in Quantum Devices,” Nanophotonics 12 (2023): 1865. 39635138 10.1515/nanoph-2023-0064 PMC11614332

[63] K. Moon , H. Park , J. Kim , et al., “Subsurface Nanoimaging by Broadband Terahertz Pulse Near‐Field Microscopy,” Nano Letters 15 (2015): 549. 25436437 10.1021/nl503998v

[64] X. G. Peralta , D. Lipscomb , G. J. Wilmink , and I. Echchgadda , “Terahertz Spectroscopy of Human Skin Tissue Models with Different Melanin Content,” Biomedical Optics Express 10 (2019): 2942. 31259064 10.1364/BOE.10.002942 PMC6583360

[65] E. A. A. Pogna , L. Viti , A. Politano , M. Brambilla , G. Scamarcio , and M. S. Vitiello , “Mapping Propagation of Collective Modes in Bi_2_Se_3_ and Bi_2_Te_2.2_Se_0.8_ Topological Insulators by Near‐Field Terahertz Nanoscopy,” Nature Communications 12 (2021): 6672. 10.1038/s41467-021-26831-6 PMC8602307 34795216

[66] R. Jing , Y. Shao , et al., “Terahertz Response of Monolayer and Few‐Layer WTe_2_ at the Nanoscale,” Nature Communications 12 (2021): 5594. 10.1038/s41467-021-23933-z PMC8458490 34552072

[67] W. A. Benalcazar , T. Li , and T. L. Hughes , “Quantization of Fractional Corner Charge in C_n_‐Symmetric Higher‐Order Topological Crystalline Insulators,” Physical Review B 99 (2019): 245151.

[68] F. Schindler , M. Brzezińska , W. A. Benalcazar , et al., “Fractional Corner Charges in Spin‐Orbit Coupled Crystals,” Physical Review Research 1 (2019): 033074.

[69] R. Takahashi , T. Zhang , and S. Murakami , “General Corner Charge Formula in Two‐Dimensional C_n_‐Symmetric Higher‐Order Topological Insulators,” Physical Review B 103 (2021): 205123.

[70] D. Zhang , M. Shi , T. Zhu , D. Xing , H. Zhang , and J. Wang , “Topological Axion States in the Magnetic Insulator MnBi_2⁢_Te_4_ with the Quantized Magnetoelectric Effect,” Physical Review Letters 122 (2019): 206401. 31172761 10.1103/PhysRevLett.122.206401

[71] I. L. Medintz , H. T. Uyeda , E. R. Goldman , and H. Mattoussi , “Quantum Dot Bioconjugates for Imaging, Labelling and Sensing,” Nature Materials 4 (2005): 435. 15928695 10.1038/nmat1390

[72] L. Li , Y. Yu , G. J. Ye , et al., “Black Phosphorus Field‐Effect Transistors,” Nature Nanotechnology 9 (2014): 372. 10.1038/nnano.2014.35 24584274

[73] S. Jiang , J. Shan , and K. F. Mak , “Electric‐Field Switching of Two‐Dimensional van der Waals Magnets,” Nature Materials 17 (2018): 406. 29531370 10.1038/s41563-018-0040-6

[74] J. L. Collins , A. Tadich , W. Wu , et al., “Electric‐Field‐Tuned Topological Phase Transition in Ultrathin Na_3_Bi,” Nature 564 (2018): 390. 30532002 10.1038/s41586-018-0788-5

[75] X. Qian , J. Liu , L. Fu , and J. Li , “Quantum Spin Hall Effect in Two‐Dimensional Transition Metal Dichalcogenides,” Science 346 (2014): 1344. 25504715 10.1126/science.1256815

