# Supplementary material for: Electric‐Field Control of Terahertz Response via Spin‐Corner‐Layer Coupling in Altermagnetic Bilayers
Source: Adv Sci (Weinh). 2026 Jan 25;13(19):e23285. doi: 10.1002/advs.202523285 (PMC13045405; doi:10.1002/advs.202523285)
Supplement: Supplementary file 1 — Supporting File: advs74046‐sup‐0001‐SuppMat.pdf. [file ADVS-13-e23285-s001.pdf]

Supporting Information for "Electric-Field Control of Terahertz Response via Spin-Corner-Layer Coupling in Altermagnetic Bilayers"

Jianhua Wang, Yilin Han, Shifeng Qian, Zhenxiang Cheng, Wenhong Wang, Zhi-Ming Yu, Xiaotian Wang**

J. Wang, W. Wang
School of Material Science and Engineering, Tiangong University, Tianjin 300387, China
J. Wang, Z. Cheng, X. Wang
Institute for Superconducting and Electronic Materials, Faculty of Engineering and Information Sciences, University of Wollongong, Wollongong 2500, Australia
Email:xiaotianw@uow.edu.au
Y. Han, Z.-M. Yu
Key Lab of Advanced Optoelectronic Quantum Architecture and Measurement (MOE), Beijing Key Lab of Nanophotonics & Ultrafine Optoelectronic Systems, and School of Physics, Beijing Institute of Technology, Beijing 100081, China
S. Qian
Anhui Province Key Laboratory for Control and Applications of Optoelectronic Information Materials, Department of Physics, Anhui Normal University, Wuhu, Anhui 241000, China
Email:qiansf@ahnu.edu.cn

S1 Computational Methods

First-principles calculations were conducted using the Vienna ab initio simulation package (VASP) [1]. The exchange-correlation energy was treated within the Perdew-Burke-Ernzerhof (PBE) generalized gradient approximation (GGA). The projector-augmented wave pseudopotentials [2] were used to simulate the interaction between ions and valence electrons. A plane-wave cutoff energy of 500 eV was used for all calculations. The electronic self-consistent loop was considered converged when the energy change was less than 1×10^{-6} eV on a 7×7×1 Monkhorst-Pack k-mesh. For structural relaxation, the Hellmann-Feynman forces on each atom were converged to within 0.01 eV/Å. The DFT-D2 method [3] was used to account for the van der Waals (vdW) interactions. To account for the strong correlation effects of Ni-3d orbitals, the GGA+U approach [4] was adopted with an effective Hubbard U parameter of 3.5 eV. The discrepancies in the band structures and band gaps between our work and Ref. [5] (see **Figure S2**) arise from the fact that the Hubbard U parameter was not considered in their study. A 20 Å-thick vacuum layer was used to ensure decoupling between neighboring slabs. The irreducible representations of electronic states from DFT results were calculated using the irvsp code [6]. Finally, Wannier tight-binding model was constructed with the Wannier90 package [7], which was subsequently used to calculate the energy spectrum of the nanodisk.

Table S1: Absorption peak frequency and transition dipole moment under various electric-field strengths.

Electric field strength (eV/Å)	Frequency (THz)	Transition dipole moment (eÅ)
0.0008	0.0855	[-12.30266-0.1745i, 19.91592+0.54942i, -0.48284-0.01003i]
	0.0933	[-22.41527-1.18444i, 0.03377-0.02913i, 0.54877+0.04533i]
0.0012	0.1457	[7.65745-4.80808i, 12.89921-8.10505i, 0.27161-0.72739i]
	0.1644	[-17.24431-4.42470i, 0.09621-0.00100i, 0.51114+0.03392i]
-0.0008	0.0766	[1.79463-12.17252i, 3.12666-20.42326i, 0.07834-0.45217i]
	0.0986	[19.20091-1.02116i, -0.00444-0.02934i, -0.47086+0.00748i]
-0.0012	0.1306	[-10.55177+1.83854i, 17.49028-3.14069i, -0.639+0.30404i]
	0.1368	[-20.08427+4.59056i, -0.10167+0.04037i, 0.55045-0.15403i]

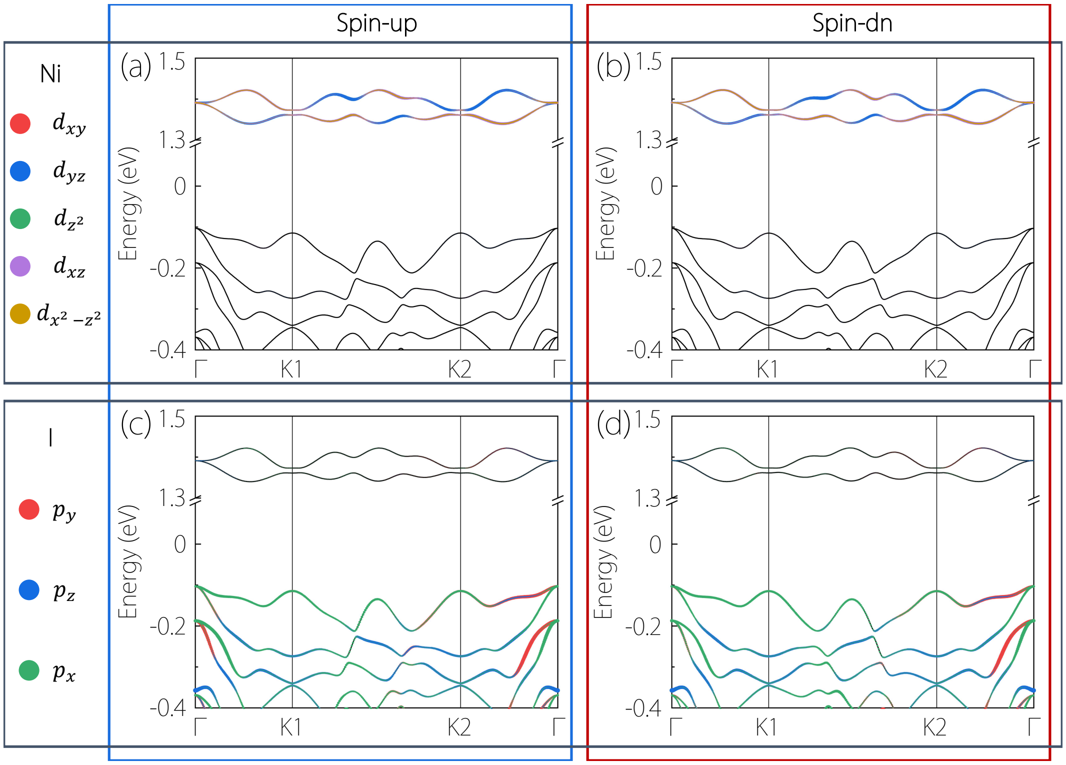

Figure S1: (a, c) The orbital-resolved band structures of altermagnetic bilayer $NiZrI_6$ for spin-up. (b, d) The orbital-resolved band structures of altermagnetic bilayer $NiZrI_6$ for spin-down.

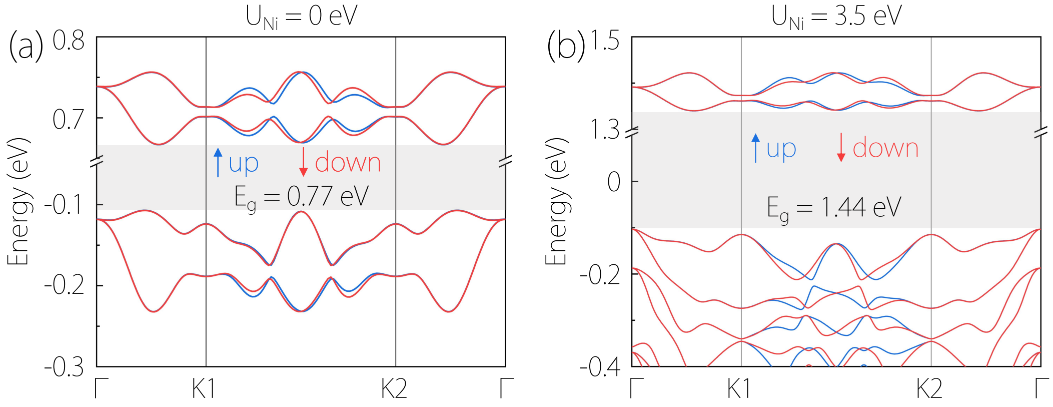

Figure S2: (a) and (b) show the band structures of altermagnetic bilayer $NiZrI_6$ with different U values, respectively.

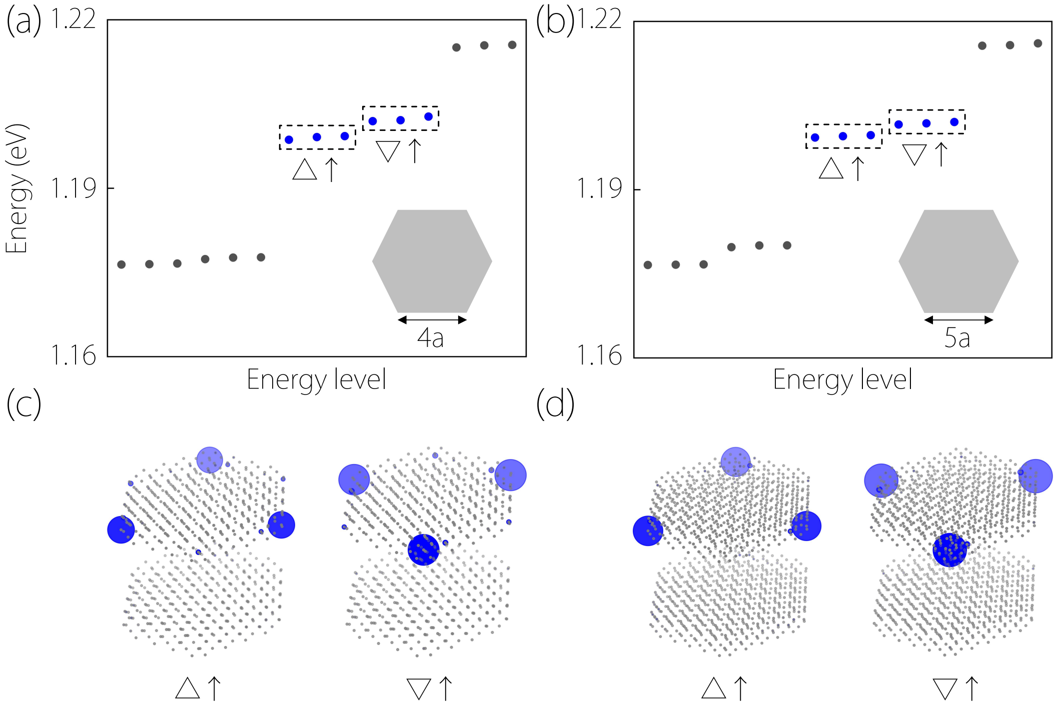

Figure S3: (a, b) Energy spectra of hexagonal nanodisks with side lengths of 4a and 5a, respectively. (c, d) Spatial distributions of the corresponding corner states.

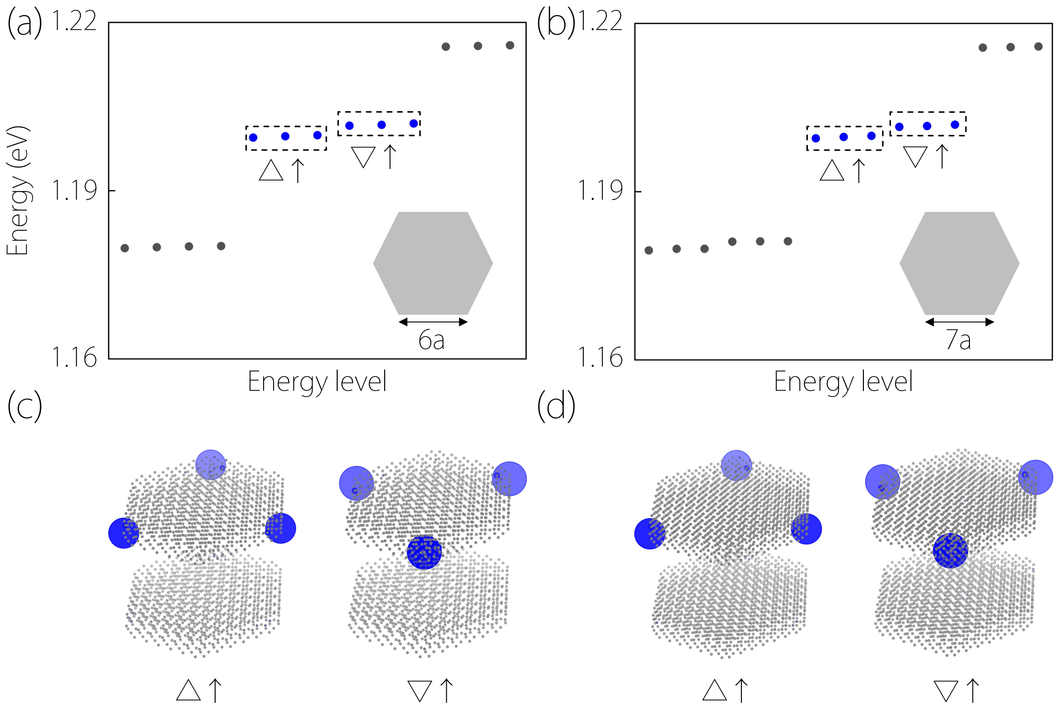

Figure S4: (a, b) Energy spectra of hexagonal nanodisks with side lengths of 6a and 7a, respectively. (c, d) Spatial distributions of the corresponding corner states.

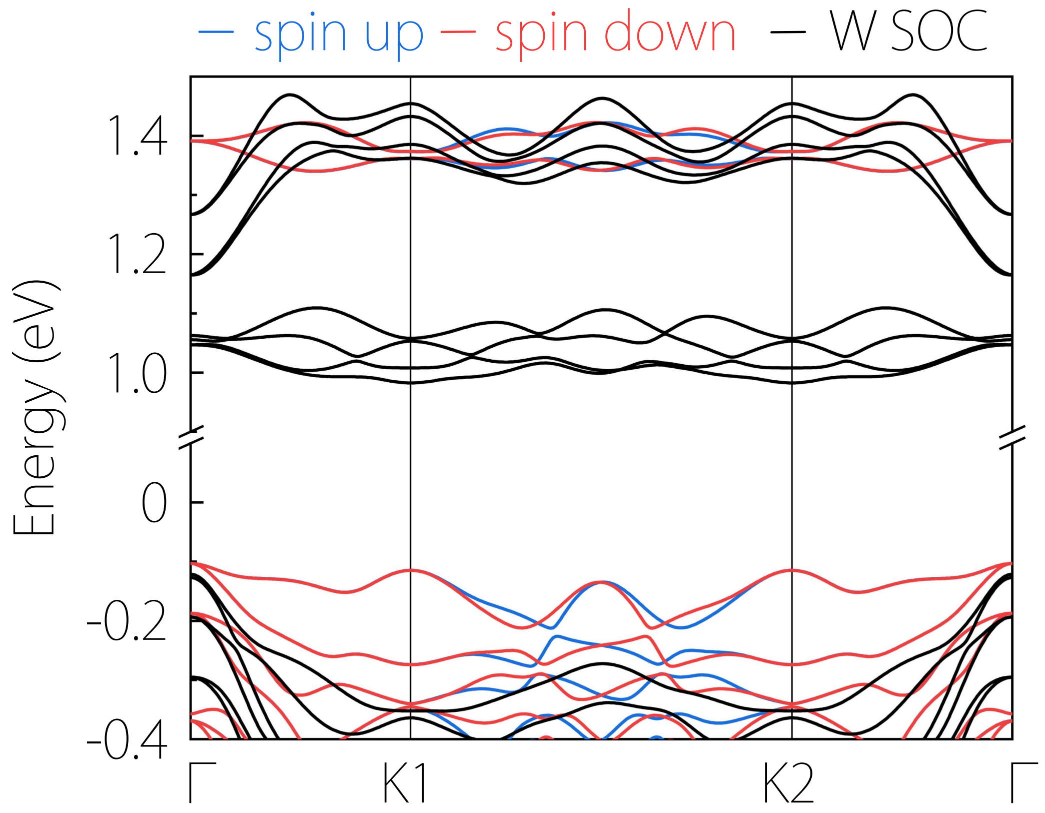

Figure S5: Band structure comparison without and with SOC.

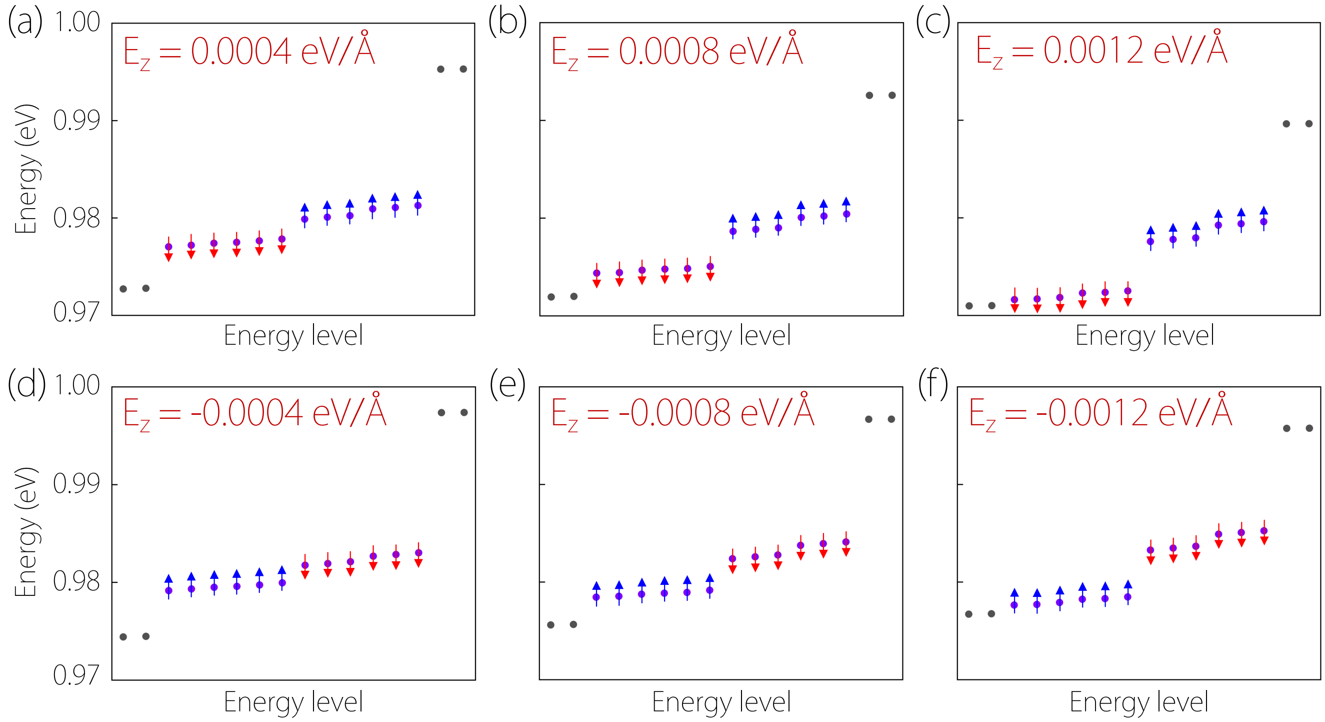

Figure S6: (a-f) Energy spectrum for $NiZrI_6$ nanodisks with SOC under E_z = 0.0004, 0.0008, 0.0012, -0.0004, -0.0008, and -0.0012 eV/Å, respectively.

References

[1] J. P. Perdew, K. Burke, M. Ernzerhof, Phys. Rev. Lett. 1996, 77, 3865.

[2] P. E. Blöchl, Phys. Rev. B 1994, 50, 17953.

[3] S. Grimme, J. Comput. Chem. 2006, 27, 1787.

[4] V. I. Anisimov, J. Zaanen, O. K. Andersen, Phys. Rev. B 1991, 44, 943.

[5] B. Pan, P. Zhou, P. Lyu, H. Xiao, X. Yang, L. Sun, Phys. Rev. Lett. 2024, 133, 166701.

[6] J. Gao, Q. Wu, C. Persson, Z. Wang, Comput. Phys. Commun. 2021, 261, 107760.

[7] A. A. Mostofi, J. R. Yates, Y.-S. Lee, I. Souza, D. Vanderbilt, N. Marzari, Comput. Phys. Commun. 2008, 178, 685.
